# Uses of Health Care System Medical Care Services by Athletes After Injury at the High School Level

**DOI:** 10.1111/josh.13255

**Published:** 2022-10-20

**Authors:** Garrett S. Bullock, John F. Mobley, John M. Brooks, Mitchel J. Rauh, R. Gil Gilliland, Michael J. Kissenberth, Ellen Shanley

**Affiliations:** ^1^ Department of Orthopaedic Surgery & Rehabilitation, Wake Forest School of Medicine, Winston‐Salem, NC; Centre for Sport, Exercise and Osteoarthritis Research Versus Arthritis University of Oxford Oxford UK; ^2^ University of South Carolina School of Medicine Columbia SC; ^3^ South Carolina Center for Effectiveness Research in Orthopaedics University of South Carolina Columbia SC; ^4^ College of Health and Human Services Sand Diego State University San Diego CA; ^5^ Steadman Hawkins Clinic of the Carolinas Greenville SC; ^6^ Steadman Hawkins Clinic of the Carolinas Greenville SC; ^7^ University of South Carolina Center for Rehabilitation and Reconstruction Sciences, Greenville, SC; ATI Physical Therapy Greenville SC

**Keywords:** socioeconomic status, health care access, emergency department, physical therapy

## Abstract

**Background:**

Health care utilization can vary by age group, geographic location, and socioeconomic status (SES). A paucity of information exists regarding the availability and utilization of medical care by injured scholastic athletes. The purpose of this study was to describe and compare injuries and health care service utilization by school SES over an academic year.

**Methods:**

Injury and health care service data was collected from 1 large school district. Percentage of free and reduced lunch (FRPL) for each school was calculated to stratify schools into high (<50% FRPL) and low (≥50.1% FRPL) SES groups. Incidence proportion and relative risk (RR) with 95% confidence intervals (95% CI) were calculated.

**Results:**

About 1756 injuries were reported among over 7000 participating athletes from 14 high schools. Similar injury incidence proportions were reported between high and low SES schools (RR = 1.10 [1.00–1.20]). Athletes from low SES schools were twice (RR = 2.01 [1.21–3.35]) and over three (RR = 3.42 [1.84–6.55]) times more likely to receive emergency and physical therapy care. SES was not associated with the use of physician, imaging, or surgery services.

**Implications for School Health, Policy, and Equity:**

School medical providers and administrators should have ready and provide a list of trusted outside primary care and specialty providers that have experience in sports medicine. They should also enquire and follow up on which outside provider the high school athlete will seek care when referring out to outside providers.

**Conclusions:**

Injury incidence was similar between high and low SES schools. However, athletes from low SES high schools were over 2‐fold more likely to use emergency department services. Understanding factors influencing health care services choice and usage by student athletes from different socioeconomic backgrounds may assist sport medicine clinicians in identifying barriers and potential solutions in improving time to health restoration, athlete outcomes, and health care monetary burden.

The National Federation of State High Schools reports that over 7 million athletes participate in high school sports in the United States each year.^1^ Sport participation has been documented to promote many positive benefits including increased physical fitness, improvement in academic performance, and a decrease in corrosive behaviors during high school.^2^ With participation in organized sports, some adverse effects have been noted such as musculoskeletal injuries and the need for medical treatment. Of the documented 7 million athletes, approximately 2 million are injured each year, with 500,000 receiving physician services for an injury.^3^, ^4^ Without proper injury treatment, student athletes may be unable to return to play and may experience academic, health, and social challenges.^5^

Health care utilization is a commonly studied aspect of medical care within the United States. Factors such as location, ethnicity, age, and socioeconomic status (SES) have been illustrated to strongly affect both the utilization rate and the type of care accessed by patients.^6^, ^7^ Prior studies have shown decreased health care utilization and an increased likelihood of seeking out emergency room services rather than primary care consultations for health concerns among rural and lower socioeconomic populations.^6^, ^7^ However, these studies have largely been reported in adult populations for a variety of medical conditions. Multiple factors in younger athletic populations, such as academic identity and achievement, athletic identity, SES, educational level and race, have been postulated as potential reasons for avoiding or delaying treatment for injuries such as concussions with equivocal results.^8^, ^9^, ^10^ In addition, uninsured young adults have a higher likelihood of being refused treatment for significant athletic injuries.^11^, ^12^ At the high school level, decreased access to athletic trainers and physical therapists has been noted in lower SES schools.^13^ Currently, a notable gap in knowledge exists in assessing the relationships between student athlete SES, access to sports medicine services and access to a broad range of medical care services often required by injured high school athletes.

Previous authors have explored the influence of SES on the access and availability of on‐site secondary school athletic trainers and sports medicine staff.^12^, ^14^ Student athletes from low socioeconomic households may have decreased resources including cultural and social capital and health literacy, which may inhibit students' educational success and health care choices.^12^, ^14^, ^15^ One measure to assess SES is free and reduced lunch.^16^, ^17^ Free and reduced lunch (FRPL) is a measure that has been used extensively within educational research,^16^, ^17^ and has been found to be a better measure of educational disadvantage compared to measures such as household income.^16^ Access, utilization, and timeliness of referred health care services in schools with full‐time athletic trainers has yet to be explored within public health. Additionally, there is little understanding how athletes from schools of different SES, categorized by percent eligibility to FRPL, utilized outside health care services when provided with similar sports medicine care and hours.

Currently, there is uncertainty if health care service referrals and utilization following a high school athletic injury is different according to socioeconomic backgrounds. Understanding these potential health care differences can aid sports medicine clinicians in identifying barriers and potential public health interventions to improve athlete outcomes following injury. Therefore, the purpose of this study was to describe and compare injuries and health care service utilization by school SES over an academic year. It was hypothesized that athletes at lower SES high schools would use a lower ratio (ie, proportion) of health care services compared to athletes from higher SES high schools.

## METHODS

### Study Design

This ecological cohort study was conducted using de‐identified participation, injury, and service data collected from July 1, 2018 to June 30, 2019 from 1 large school district in South Carolina. The school district provides care in collaboration with the regional hospital system's athletic training staff. The school district is comprised of 14 high schools and each school has a minimum of 1 full‐time equivalent athletic trainer and 1 sport physician based on athletic population, not high school SES. All athletic trainers were educated and disseminated the same injury prevention education at the onset of each academic school year. This study was approved by the University Institutional Review Board.

### Inclusion and Exclusion Criteria

Athlete records were included if the documented athlete was deemed eligible for participation by the state high school league, fully rostered to a minimum of 1 scholastic athletic team, and participated during the 2018‐2019 school year. Exclusion criteria consisted of medical records that were outside the 2018‐2019 academic year and or from athletes who attended a high school that was in a school district outside of the prescribed county.

### Injury Definition

An injury was defined as an injury to a tendon, ligament, nerve, muscle, or bone that occurred during any high school team sponsored activity or event,^18^ required medical attention from the full‐time equivalent athletic trainer or team physician, and resulted in at least 1 day missed from competition.^19^ Injuries were defined by the Orchard Sports Injury Classification system.^20^ Injury types were further classified according to the previous work of Powell & Barber Foss.^3^ Injury types were classified as general trauma (eg, contusions, wounds, cramps, acute inflammation), neurotrauma (eg, concussions, seizures), sprains, strains, fractures, musculoskeletal conditions (eg, ilio‐tibial band syndrome, plantar fasciitis, cartilage damage), general stress (eg, skin conditions, asthma, undetermined pain), instability (eg, dislocations and subluxations), and illness.

### Injury and Heath Service Tracking

A previously validated^21^ standardized injury and health service process was utilized. Services within and without the county health care system were joined, with <1% of all health care services coming from outside the county health care system. Information included in the injury files included participation status (by school and team), injury occurrence (by date of service, mechanism of injury, body part, injury and event type), and all medical care (by location, date of service, service type, provider type, and provider) documented by the school's athletic trainer in the secure database.^21^ For further detail, please refer to Appendix S1.

### Health Care Treatment

Treatment was provided according to standard care programs established by the sports medicine department. The assigned full‐time equivalent athletic trainer provided initial musculoskeletal and rehabilitative care within their designated scope of practice whereas more specialized care was accessed through outside providers. Care was provided on site or through an outside referral to an appropriate service provider, as needed. Outside services were recommended by the athletic trainer or team physician if further care was needed, however, provider choice and location were independently selected by the athlete or family. High school athletic trainer experience was similar between high (mean: 8 [range: 4–14]) and low socioeconomic (mean: 13 [range: 4–15]) schools.

### School Socioeconomic Status

For the objective to measure the SES for each athlete in our study, the Family Educational Rights and Privacy Act (FERPA) restricted our ability to obtain SES for each athlete. Thus, following the approach often used in health services research, we used an “area‐wide” proxy for individual SES.^22^, ^23^ Each athlete was assigned a SES designation using the percentage of students receiving FRPL at their high school. Student FRPL eligibility is determined by household families' income. Families that are below 130% of the United States federal poverty level are eligible for free lunch, while families between 130% and 185% of the federal poverty level are eligible for reduced priced lunch. Reduced price meals cannot be priced higher than 30 cents for breakfast and 40 cents for lunch.^24^ Based on the recommendations of the National Education Foundation, school SES was stratified by high SES (percentage FRPL ≤50%) and low SES (FRPL ≥50.1%).^24^ We are aware that using an aggregate proxy for SES will tend to bias estimates of the effect of SES on health care treatment toward zero^22^, ^23^, ^25^, ^26^ so that our estimates should be interpreted as lower bounds of the true effects.

### Data Reduction

Athlete injury and health care service files were merged into a single file using Microsoft SQL Server, joined by the common athlete and injury identification numbers. Data were collected for each athlete who sustained each injury to measure athlete sex, year of high school, injury sustained, sport, and school SES.

To make meaningful comparisons between sports, sport played was collapsed into collision, field and court sport, overhead, and individual.^27^

### Statistical Analysis

Participation, injury, and health care services were reported as count and percentages. Missing data was low (overall missing: 0.5%; sex: <0.1%; sport: 0.1%; state: 0%; high school: 0%; body part: 0.2%; date: 0.1%; school year: 0%), thus complete case analyses were performed. Incidence proportion (with 95% confidence intervals [95% CI]) per 100 of injury and health services per 100 athletes were calculated. Incidence proportion (also known as cumulative incidence) of health service was also calculated per injury.^28^ Risk ratios (RR) (with 95% CI) were also used to examine service utilization between groups. Poisson regressions with 95% CI were used to determine the likelihood of an athlete receiving health care services (emergency room services, physician consultation at office, imaging, surgery or physical therapy) between athletes at high and low socioeconomic schools. Poisson models have demonstrated improved likelihood assessment of an event (ie, injury) when the outcome is common (ie, >10%) compared to logistic regressions.^29^ Models were controlled at the individual (ie, athlete) and cluster (ie, school) level. Confounders controlled for included age, sex, school, and sport, and an offset of athlete participation. The sensitivity analysis included (1) only severe injuries comprising anterior cruciate ligament tears, neurotrauma, dislocations, and fractures; (2) stratifying by gender; (3) only high school level data. All analyses were performed in R version 3.5.1 (R Core Team, 2013). R: A language and environment for statistical computing. The epitools package was used to calculate incidence proportion ratios.

## RESULTS

### General Findings

About 1756 injuries were reported among over 7000 participating athletes from 14 high schools (Table 1). No differences were observed in injury incidence between schools (Table 2). Athletes from low socioeconomic schools were almost 30% more likely to sustain a sprain, 50% more likely to sustain a fracture, and over 2 times more likely to sustain an anterior cruciate ligament tear compared to athletes from high socioeconomic schools (Table 3).

**Table 1:** High School Athlete Descriptive Statistics

	All Schools	High SES (≤50% FRPL)	Low SES (≤50.1 +% > FRPL)
Schools (n)	14	8	6
Participation (n)	7393	4993	2400
Injuries (n)	1756	1140	616
Health care services (n)	864	520	344
Number of days to return to sport*	2 (1, 8)	1 (1, 10)	2 (1, 11)
Injury by sex (% female)	30%	32%	28%
Injury by sport (%)			
Collision	47%	46%	49%
Field and court	33%	33%	33%
Overhead	7%	7%	7%
Individual	13%	14%	12%

FRPL, free or reduced priced lunch; SES, socioeconomic status.

* Reported as median and interquartile range.

**Table 2:** Sports Injury and Health Care Services by School Socioeconomic Status

	High SES (≤50% FRPL)	Low SES (≥50.1 +% > FRPL)	Risk Ratio
Injuries/100 athletes	22.8 (95% CI: 21.7–24.0)	25.6 (95% CI: 23.9–27.4)	1.10 (95% CI: 1.00–1.20)
Services/100 athletes	10.4 (95% CI: 9.6–11.3)	14.3 (95% CI: 12.9–15.7)	1.33 (95% CI: 1.17–1.51)
Services/100 injuries	45.6 (95% CI: 42.7–48.5)	55.8 (95% CI: 51.9–59.8)	1.14 (95% CI: 1.02–1.28)

FRPL, free or reduced priced lunch; SES, socioeconomic status.

**Table 3:** Injury Type by School Socioeconomic Status

	Total (n = 7393)		High SES (n = 4993)		Low SES (n = 2400)		
Injury	Injuries^†^	IP^‡^	Injuries^†^	IP^‡^	Injuries^†^	IP^‡^	Risk Ratio*	(95% CI)
Sprain	506	6.8	311	6.3	195	8.1	1.28^§^	(1.08–1.52)
Strain	310	4.2	214	4.3	96	4.0	0.94	(0.74–1.18)
Neurotrauma	238	3.2	162	3.2	76	3.2	0.98	(0.75–1.28)
Fractures	164	2.2	95	1.9	69	2.9	1.50^§^	(1.10–2.03)
ACL tears	23	0.3	11	0.2	12	0.5	2.26^§^	(1.01–5.12)
Instability	56	0.8	39	0.8	17	0.7	0.91	(0.78–1.60)
Musculoskeletal conditions	38	0.5	20	0.4	18	0.8	1.87	(0.98–3.52)
General trauma	367	5.0	251	5.0	106	4.4	0.88	(0.71–1.10)
General stress	47	0.6	26	0.5	21	0.9	1.67	(0.94–2.97)
Illness	17	0.2	11	0.2	6	0.3	1.13	(0.42–3.06)

IP, incidence proportion; SES, socioeconomic status.

Low socioeconomic status is defined of proportion of school free or reduced lunch of ≥50.1%.

High socioeconomic status is defined of proportion of school free or reduced lunch of 0%‐50.0%.

* Risk ratio is comparing high and low socioeconomic status schools.

^†^ Number of scholastic musculoskeletal injuries per injury type.

^‡,§^ These are shown with the 95% CI.

### Risk Ratio Comparison Between Athletes From High and Low Socioeconomic Schools

Athletes from low SES schools were almost twice (RR: 1.93; 95% CI: 1.18‐3.14) as likely to use emergency room services following injury compared to athletes from high SES schools (Table 4). Athletes from low SES schools were nearly over 3 (RR: 3.35; 95% CI: 1.80‐6.22) times more likely to use physical therapy services following injury compared to athletes from high SES schools. No differences were observed for physician, imaging, and surgery services between athletes from different socioeconomic schools.

**Table 4:** Utilization by School Socioeconomic Status and Health Care Service Type

	Total (n = 7393)			High SES (n = 4993)			Low SES (n = 2400)			
Services	N	Injuries*	IP^†^	N	Injuries*	IP^†^	N	Injuries*	IP^†^	Risk Ratio	(95% CI)
Physician visits	587	1756	33.4	376	1140	32.9	211	616	34.2	1.03	(0.89–1.19)
Emergency visit	62	1756	3.5	30	1140	2.6	32	616	5.2	1.93^‡^	(1.18–3.14)
Imaging	104	1756	5.9	62	1140	5.4	42	616	5.2	1.23	(0.85–1.81)
Surgery	68	1756	3.9	37	1140	3.2	31	616	4.3	1.51	(0.96–2.43)
Physical therapy	43	1756	2.4	15	1140	1.3	28	616	4.5	3.35^‡^	(1.80–6.22)

IP, incidence proportion; N, number; SES, socioeconomic status.

Low socioeconomic status is defined of proportion of school free or reduced lunch of ≥50.1%.

High socioeconomic status is defined of proportion of school free or reduced lunch of 0–50.0%.

* Injuries = Scholastic musculoskeletal injuries occurring in school sponsored practice or game.

^†^ Incidence Proportion = Service incidence per 100 injuries.

^‡^ Significant difference between Low vs. High socioeconomic groups.

### Relative Risk Analyses Comparing Health Care Service Utilization Between Athletes From High and Low Socioeconomic Schools

After adjusting for confounders, athletes participating at low socioeconomic schools were over three times more likely (unadjusted: RR: 3.49, 95% CI: 3.10‐3.94, p = 0.043; adjusted: RR: 3.42, 95% CI: 1.84‐6.55, p < 0.001) to utilize physical therapy care compared to athletes from high socioeconomic school. Athletes participating at low socioeconomic schools were 2 times more likely (unadjusted: RR: 1.97, 95% CI: 1.18‐3.29, p = 0.008; adjusted: RR: 2.01, 95% CI: 1.21‐3.35, p = 0.007) to receive care at an emergency department compared to athletes from high socioeconomic schools. School SES was not associated with the use of physician (unadjusted: RR: 1.04, 95% CI: 0.88‐1.23, p = 0.660; adjusted: RR: 1.03, 95% CI: 0.87‐1.22, p = 0.706), imaging (unadjusted: RR: 1.23, 95% CI: 0.81‐1.86, p = 0.316; adjusted: RR: 1.23, 95% CI: 0.81‐1.86, p = 0.706) or surgery (unadjusted: RR: 1.54, 95% CI: 0.94‐2.51, p = 0.080; adjusted: RR: 1.50, 95% CI: 0.91‐2.44, p = 0.101) services.

### Sensitivity Analyses

Sensitivity analyses demonstrated similar adjusted results for use of physical therapy (RR: 2.83, 95% CI: 1.13–7.10, p = 0.027), physician (RR: 0.89, 95% CI: 0.70–1.14, p = 0.393), and imaging (RR: 0.91, 95% CI: 0.47–1.70, p = 0.779). Athletes from low socioeconomic schools did not demonstrate adjusted difference in emergency department visits for severe injuries (RR: 1.26, 95% CI: 0.57–2.66, p = 0.558) compared to athletes at high socioeconomic schools. Athletes from low socioeconomic schools were almost two times more likely to undergo surgery for severe injuries (RR: 1.92, 95% CI: 1.01, 1.36, p = 0.033) compared to athletes at high socioeconomic schools. Females and males demonstrated similar results for health care services used for of physical therapy (female: RR: 1.70, 95% CI: 0.57, 5.07, p = 0.341; male: 4.80, 95% CI: 2.1, 10.8, p < 0.001), physician (female: RR: 0.94, 95% CI: 0.68, 1.31, p = 0.722; male: RR: 1.07, 95% CI: 0.88, 1.30, p = 0.497), imaging (female: RR: 1.55, 95% CI: 0.74, 3.25, p = 0.249; male: RR: 1.13, 95% CI: 0.69, 1.86, p = 0.621), and surgery (female: RR: 0.99, 95% CI: 0.30, 3.24, p = 0.989; male: RR: 1.67, 95% CI: 0.97, 2.85, p = 0.063). Female emergency department care usage was similar compared to the primary analyses (females: RR: 3.37, 95% CI: 1.27, 8.94, p = 0.014). Males emergency department care point estimate was similar to the primary analyses; however, the CIs passed one (males: RR: 1.67, 95% CI: 0.92, 3.04, p = 0.093). Including on high school level data demonstrated similar results for all health care services used between low and high socioeconomic schools.

## DISCUSSION

The main findings of our study were that the number of injuries per 100 athletes were similar regardless of the school SES. However, athletes at lower socioeconomic schools utilized a higher ratio of overall outside health care services, specifically emergency department and physical therapy services, compared to athletes at higher SES schools.

These data demonstrated similar injury reporting compared to previous literature, including prevalence of severe injuries,^30^ fractures,^31^ injury type,^3^ providing generalizability of these injury reporting. Despite issuing similar in‐school health care provider access and referral services, athletes from low socioeconomic schools used a greater ratio of emergency room visits compared to athletes at high socioeconomic schools. Previous research has observed that lower socioeconomic families have less access to an established primary care provider.^32^, ^33^, ^34^ This was supported within the severe injury sensitivity analyses. Severe injury emergency department service utilization ratios were similar, suggesting that athletes from lower socioeconomic schools used emergency department services at a greater incidence proportion for nonsevere (ie, lower severity) injury care compared to athletes from high socioeconomic school. However, when stratifying by gender, females demonstrated greater relative risk compared to males. This sensitivity analyses had decreased power, and should be interpreted with caution. However, potential gender discrepancies require further enquiry in future studies. Emergency department utilization in youth and adolescents is multi‐factorial and often include access to physician care, insurance status, transportation difficulties, and cultural and language barriers.^35^ Other potential influences include injury reporting,^36^ athlete and caregiver literacy,^37^, ^38^ and health care navigation.^39^ These dynamics seem to increase the emergency department use for both initial and follow up care and often create a delay in receiving care.^35^ Using emergency care services for an initial diagnosis has the potential to delay access to a provider who can offer specialty treatment for the athlete's condition and may influence the timeline for recovery from injury.^32^, ^33^, ^34^, ^35^, ^40^

The use of different health care pathways outside of in school health care services for different socioeconomic groups is concerning. Athletes at all high schools in this study were under the care of full‐time equivalent athletic training and physician care during school sponsored athletic activities. These sport health care resources would allow for proper athletic injury identification and referral services. However, despite the consumption of similar imaging and surgical health care services between socioeconomic groups, the navigation to these endpoint imaging and surgical health care services were dissimilar. In office physician consultations offers streamlined access to surgical services compared to emergency department services in terms of sport related injuries.^40^ Longer time to orthopedic and sport physician consultations following athlete injury has been related to decreased athlete outcomes, increased time to return to sport, and increased monetary health care burden.^32^, ^33^, ^34^, ^40^ Future research is needed to understand the complex multifactorial cultural, educational, and logistical barriers inhibiting improved access and choice of proper health care expertise following high school athletic injuries in different socioeconomic schools.

Athletes from lower socioeconomic schools demonstrated a greater ratio of physical therapy services compared to athletes from higher socioeconomic schools. Physical therapy services may have been accessed due to the greater incidence proportion of anterior cruciate ligament tears and fractures in athletes from lower socioeconomic schools. Further, within the sensitivity analysis, athletes from lower socioeconomic schools underwent surgery at a greater ratio for severe injuries compared to athletes from higher socioeconomic schools. Physical therapy is prescribed following orthopedic surgery and severe injuries to return to previous function and athletic levels.^41^ These differences in physical therapy care utilization may explain these injury and surgery differences. However, this discrepancy is the beyond the scope of this study and further research is required to understand the relationship between SES and high school athlete physical therapy care use.

### Clinical Implications

These results illuminate potential clinical implications. Sports medicine clinicians that cover high school athletes on campus or treat them in a training room should enquire with the athlete and family about clinical health service choice at initial health care referral. As suggested from these results, athletes that participate at different socioeconomic schools, utilize different ratios of emergency department services, despite similar sports medicine coverage. As stated previously, emergency department use as a primary provider is dependent on a multitude of factors, and can result in longer periods to seeking consultation of a sports medicine specialist, which can impede athlete health.^32^, ^33^, ^34^, ^40^ At the initial injury and health care referral, sports medicine clinicians should enquire about the athlete's potential physician and physical therapy choices, and how they can affect their return to sport. Further, clinicians can potentially provide suggestions for health care providers, if the athlete and their family do not have a preferred provider. The clinician should follow up on this enquiry with the athlete at subsequent examinations and potentially contact the chosen health care provider for handling of care.

## Implications for School Health Policy, Practice, and Equity

This ecological cohort study included one large school district in South Carolina. The school district provides care in collaboration with the regional hospital system's athletic training staff. The school district is comprised of 14 high schools and each school has a minimum of 1 full‐time equivalent athletic trainer and 1 sport physician based on athletic population, not high school SES. Treatment was provided according to standard care programs established by the sports medicine department. The assigned full‐time equivalent athletic trainer provided initial musculoskeletal and rehabilitative care within their designated scope of practice whereas more specialized care was accessed through outside providers. Care was provided on site or through an outside referral to an appropriate service provider, as needed. Outside services were recommended by the athletic trainer or team physician, however, provider choice and location were independently selected by the athlete or family. At the time of data collection, potential physician and physical therapy choices, and enquiry with the athlete on going to said provider, were not a part of the provider process. This study found that injury incidence were similar between high schools; however, lower socioeconomic high schools used a higher ratio of outside health care services, most noticeably emergency department services. These findings have implications for school policies, equity, and clinical practice which can impact athlete health.

- Have ready and provide a list of trusted outside primary care and specialty providers that have experience in sports medicine.
- Enquire which outside provider the high school athlete will seek care when referring out to outside providers.
- Follow up on the outside clinical visit when providing care to the high school athlete at the next treatment session.

### Limitations

Several important limitations in this study are noted. The athletes and families determined the choice of medical provider from whom they sought care. However, due to each school's team physician being from 1 health care organization, the majority of care was associated with a large local sports medicine group, which decreased the generalizability of these results. Care was also received outside of the county health care system, which was determined less than 1%. However, further health care utilization outside the county health care system could have been performed, without the sports medicine staff's knowledge, potentially inciting response bias. Due to the inconsistencies in recording athlete exposure in high school athletics, it was not possible to calculate athlete exposure nor injury or health care incidence or rate, decreasing the precision and clinical interpretability of these findings. However, the athlete injury and health care tracking system used in this study has previously been validated,^21^ increasing the strength and validity of the epidemiological reports. High school coaching experience was not recorded. As coaching experience may influence injury risk and referral, this may bias these results. While athletic trainers disseminated the same injury prevention education to all high schools, adherence and further injury prevention programs and training may have been performed differently at individual high schools and sports, potentially biasing these injury results. However, as injury proportion was similar between high schools, this bias may have minimal effect. This was an ecological study, using the high school as the unit of analysis. Individual participant level studies could improve the precision in these findings.

Lastly, because of FERPA rules, we were restricted to use a high‐school level proxy measure of SES for each athlete. There are limitations concerning FRPL use in high school students, most notably underreporting of FRPL need.^42^ It is well known that using of aggregate level proxy measures of variables will tend to bias estimates on these variables toward zero, which means that our estimates of SES on health care utilization should be correctly interpreted as the lower bounds of the true effects. As such, our statistically significant results suggest that relationships exist between SES and health care utilization for injured high school athletes, but that our actual estimates should not be used to guide policy changes.

### Conclusions

Students at lower SES schools, as determined by FRPL percentage, were more likely to utilize emergency department care and physical therapy services compared to athletes at higher SES schools. Understanding factors influencing health care services choice and usage by student athletes from different socioeconomic backgrounds may assist sport medicine clinicians in identifying and ultimately dismantling barriers in improving time to health restoration, athlete outcomes, and health care monetary burden.

### Human Subjects Approval Statement

This study was approved by the University Institutional Review Board.

### Conflicts of Interest

The authors declare they have no conflicts of interest.

## Supporting information


**Appendix S1.** Supporting Information.

## References

[1] 2017–2018 high school athletics participation survey. Available at: http://nfhs.org/ParticipationStatistics. Accessed August 2, 2019.

[2] Greenleaf C , Boyer EM , Petrie TA . High school sport participation and subsequent psychological well‐being and physical activity: the mediating influences of body image, physical competence, and instrumentality. Sex Roles. 2009;61(9):714‐726.

[3] Powell JW , Barber‐Foss KD . Injury patterns in selected high school sports: a review of the 1995‐1997 seasons. J Athl Train 1999;34(3):277–284. 16558577 PMC1322923

[4] Fox CK , Barr‐Anderson D , Neumark‐Sztainer D , Wall M . Physical activity and sports team participation: associations with academic outcomes in middle school and high school students. J Sch Health. 2010;80(1):31‐37. 10.1111/j.1746-1561.2009.00454.x. 20051088

[5] Abbott W , Brownlee TE , Harper LD , Naughton RJ , Richardson A , Clifford T . A season long investigation into the effects of injury, match selection and training load on mental wellbeing in professional under 23 soccer players: a team case study. Eur J Sport Sci. 2019;19(9):1250‐1256. 30955458 10.1080/17461391.2019.1600586

[6] Blazer DG , Landerman LR , Fillenbaum G , Horner R . Health services access and use among older adults in North Carolina: urban vs rural residents. Am J Public Health. 1995;85(10):1384‐1390. 10.2105/ajph.85.10.1384. 7573622 PMC1615634

[7] Andersen R , Newman JF . Societal and individual determinants of medical care utilization in the United States. Milbank Mem Fund Q Health Soc. 1973;51(1):95‐124. 4198894

[8] Copley M , Jimenez N , Kroshus E , Chrisman SPD . Disparities in use of subspecialty concussion care based on ethnicity. J Racial Ethn Health Disparities. 2020;7(3):571‐576. 10.1007/s40615-019-00686-6. 31898059

[9] Lempke LB , Rawlins MLW , Anderson MN , Miller LS , Lynall RC , Schmidt JD . The influence of socioeconomic status and academic standing on concussion‐reporting intentions and behaviors in collegiate athletes. Health Promot Pract. 2021;22(5):649‐658. 10.1177/1524839920920289. 32443945

[10] Wayment HA , Huffman AH , Lane TS , Lininger MR . Relationship of athletic and academic identity to concussion reporting intentions. Musculoskelet Sci Pract. 2019;42:186‐192. 10.1016/j.msksp.2019.04.003. 31014920

[11] Johnson TR , Nguyen A , Shah K , Hogue GD . Impact of insurance status on time to evaluation and treatment of meniscal tears in children, adolescents, and college‐aged patients in the United States. Orthop J Sports Med. 2019;7(10):2325967119875079. 10.1177/2325967119875079. 31620487 PMC6777051

[12] Post E , Winterstein AP , Hetzel SJ , Lutes B , McGuine TA . School and community socioeconomic status and access to athletic trainer Services in Wisconsin Secondary Schools. J Athl Train. 2019;54(2):177‐181. 10.4085/1062-6050-440-17. 30398929 PMC6464297

[13] Kirchner GE , Rivers NJ , Balogh EF , et al. Does Medicaid expansion improve access to care for the first‐time shoulder dislocator? J Shoulder Elbow Surg. 2019;28(11):2079‐2083. 10.1016/j.jse.2019.07.008. 31521525

[14] Post EG , Roos KG , Rivas S , Kasamatsu TM , Bennett J . Access to athletic trainer services in California Secondary Schools. J Athl Train. 2019;54(12):1229‐1236. 10.4085/1062-6050-268-19. 31714144 PMC6922569

[15] Walpole M . Socioeconomic status and college: how SES affects college experiences and outcomes. Rev High Educ. 2003;27(1):45‐73.

[16] Domina T , Pharris‐Ciurej N , Penner A , et al. Capturing more than poverty: School free and reduced‐price lunch data and household income; 2018.

[17] Harwell M , LeBeau B . Student eligibility for a free lunch as an SES measure in education research. Educ Res. 2010;39(2):120‐131.

[18] Rauh MJ , Margherita AJ , Rice SG , Koepsell TD , Rivara FP . High school cross country running injuries: a longitudinal study. Clin J Sport Med. 2000;10(2):110‐116. 10798792 10.1097/00042752-200004000-00005

[19] Borowski LA , Yard EE , Fields SK , Comstock RD . The epidemiology of US high school basketball injuries, 2005–2007. Am J Sports Med. 2008;36(12):2328‐2335. 18765675 10.1177/0363546508322893

[20] Rae K , Orchard J . The Orchard sports injury classification system (OSICS) version 10. Clin J Sport Med. 2007;17(3):201‐204. 10.1097/JSM.0b013e318059b536. 17513912

[21] Shanley E , Thigpen CA , Chapman CG , Thorpe J , Gilliland RG , Sease WF . Athletic trainers' effect on population health: improving access to and quality of care. J Athl Train Feb 2019;54(2):124–132. doi:10.4085/1062-6050-219-17 30461294 PMC6464298

[22] Berkowitz SA , Traore CY , Singer DE , Atlas SJ . Evaluating area‐based socioeconomic status indicators for monitoring disparities within health care systems: results from a primary care network. Health Serv Res. 2015;50(2):398‐417. 10.1111/1475-6773.12229. 25219917 PMC4369215

[23] Moss JL , Johnson NJ , Yu M , Altekruse SF , Cronin KA . Comparisons of individual‐ and area‐level socioeconomic status as proxies for individual‐level measures: evidence from the mortality disparities in American communities study. Popul Health Metrics. 2021;19(1):1. 10.1186/s12963-020-00244-x. PMC7792135 33413469

[24] Statistics NCfE . Concentration of public school students eligible for free or reduced‐Price lunch. ISE‐ National Center for Education Statistics; 2021. Accessed January 28, 2021.

[25] Geronimus AT , Bound J . Use of census‐based aggregate variables to proxy for socioeconomic group: evidence from national samples. Am J Epidemiol. 1998;148(5):475‐486. 10.1093/oxfordjournals.aje.a009673. 9737560

[26] Soobader M , LeClere FB , Hadden W , Maury B . Using aggregate geographic data to proxy individual socioeconomic status: does size matter? Am J Public Health. 2001;91(4):632‐636. 10.2105/ajph.91.4.632. 11291379 PMC1446644

[27] Christopher S , Tadlock BA , Veroneau BJ , et al. Epidemiological profile of pain and non‐steroid anti‐inflammatory drug use in collegiate athletes in the United States. BMC Musculoskelet Disord. 2020;21(1):1‐9. 10.1186/s12891-020-03581-y PMC7437034 32814544

[28] Knowles SB , Marshall SW , Guskiewicz KM . Issues in estimating risks and rates in sports injury research. J Athl Train. 2006;41(2):207‐215. 16791309 PMC1472638

[29] McNutt L‐A , Wu C , Xue X , Hafner JP . Estimating the relative risk in cohort studies and clinical trials of common outcomes. Am J Epidemiol. 2003;157(10):940‐943. 12746247 10.1093/aje/kwg074

[30] Darrow CJ , Collins CL , Yard EE , Comstock RD . Epidemiology of severe injuries among United States high school athletes: 2005‐2007. Am J Sports Med. 2009;37(9):1798‐1805. 19531659 10.1177/0363546509333015

[31] Swenson DM , Henke NM , Collins CL , Fields SK , Comstock RD . Epidemiology of United States high school sports‐related fractures, 2008‐09 to 2010‐11. Am J Sports Med. 2012;40(9):2078‐2084. 22837429 10.1177/0363546512453304 PMC3852886

[32] Leyenaar JK , Shieh MS , Lagu T , Pekow PS , Lindenauer PK . Hospital and community characteristics associated with pediatric direct admission to hospital. Acad Pediatr. 2018;18(5):525‐534. 10.1016/j.acap.2017.10.002. 29111274 PMC5924569

[33] Diao K , Tripodis Y , Long WE , Garg A . Socioeconomic and racial disparities in parental perception and experience of having a medical home, 2007 to 2011‐2012. Acad Pediatr. 2017;17(1):95‐103. 10.1016/j.acap.2016.07.006. 27457406

[34] Stingone JA , Claudio L . Disparities in the use of urgent health care services among asthmatic children. Ann Allergy Asthma Immunol. 2006;97(2):244‐250. 10.1016/S1081-1206(10)60021-X. 16937759

[35] Taylor T , Salyakina D . Health care access barriers bring children to emergency rooms more frequently: a representative survey. Popul Health Manag. 2019;22(3):262‐271. 10.1089/pop.2018.0089. 30160608 PMC6555172

[36] Wallace J , Bretzin A , Beidler E , et al. The underreporting of concussion: differences between black and white high school athletes likely stemming from inequities. J Racial Ethn Health Disparities. 2021;8(4):1079‐1088. 32926391 10.1007/s40615-020-00864-x

[37] Wallace J , Affagato R , Brooke M , McAllister‐Deitrick J , Moran RN , Covassin T . Racial disparities in parent knowledge of concussion and recognition of signs and symptoms. J Safety Res. 2020;75:166‐172. 33334474 10.1016/j.jsr.2020.09.007

[38] Wallace J , Covassin T , Moran R . Racial disparities in concussion knowledge and symptom recognition in American adolescent athletes. J Racial Ethn Health Disparities. 2018;5(1):221‐228. 28389906 10.1007/s40615-017-0361-1

[39] Wallace J , Hou BQ , Hajdu K , et al. Healthcare navigation of black and white adolescents following sport‐related concussion: a path towards achieving health equity. J Athl Train. 2021;57:352‐359. 10.4085/1062-6050-0330.21 PMC9020596 35439315

[40] Hettrich CM , Zacharias A , Ortiz SF , et al. Are there racial differences between patients undergoing surgery for shoulder instability? Data from the multicenter orthopaedic outcomes network (MOON) shoulder instability group. J Shoulder Elb Surg. 2020;30(2):229‐236. 10.1016/j.jse.2020.09.043 33166646

[41] Ardern CL , Glasgow P , Schneiders A , et al. 2016 consensus statement on return to sport from the first world congress in sports physical therapy, Bern. Br J Spor Med. 2016;50(14):853‐864. 10.1136/bjsports-2016-096278 27226389

[42] Mirtcheva DM , Powell LM . Participation in the National School Lunch Program: importance of school‐level and neighborhood contextual factors. J Sch Health. 2009;79(10):485‐494. 19751310 10.1111/j.1746-1561.2009.00438.x

