# Supplementary material for: Uses of Health Care System Medical Care Services by Athletes After Injury at the High School Level
Source: J Sch Health. 2022 Oct 20;93(1):5–13. doi: 10.1111/josh.13255 (PMC10091823; doi:10.1111/josh.13255)
Supplement: Supplementary file 1 — Appendix S1. Supporting Information. [file JOSH-93-5-s001.docx]

**Appendix 1.** Injury and Health Service Tracking Further Methods

A previously validated^1^ standardized injury and health service process was utilized. Each injury file included one record per injury reported to the assigned full-time equivalent athletic trainer during the 2018-2019 academic year with each individual and injury being assigned a unique athlete and injury identification number within an encrypted database. The service file notes contained one record for each medical care treatment or service received by an injured athlete for each specific injury during the 2018-2019 school year. Services within and without the county healthcare system were joined, with <1% of all healthcare services coming from outside the county healthcare system. The services and treatments in the medical services and injury files were linked by the unique athlete and injury identification numbers present within the injury files. Information included in the injury files included participation status (by school and team), injury occurrence (by date of service, mechanism of injury, body part, injury and event type), and all medical care (by location, date of service, service type, provider type, and provider) documented by the school’s athletic trainer in the secure database.^1^

**References**

1. Shanley E, Thigpen CA, Chapman CG, Thorpe J, Gilliland RG, Sease WF. Athletic Trainers' Effect on Population Health: Improving Access to and Quality of Care. *J Athl Train.* 2019;54(2):124-132.
